# Methyltransferase like 13 promotes malignant behaviors of bladder cancer cells through targeting PI3K/ATK signaling pathway

**DOI:** 10.1515/biol-2022-0981

**Published:** 2024-12-18

**Authors:** Jun Zhang, Jiejie He, Ziyang Qiang, Junli Zhang, Fengchen Hao, Shiqi Song, Xiuying Chen, Wei Ma, Yan Li

**Affiliations:** Department of Urology Surgery, Affiliated Hospital of Qinghai University, No. 29, Tongren Road, West of the City, Xining, 810000, Qinghai, China; Department of Gynecologic Surgery, Affiliated Hospital of Qinghai University & Affiliated Cancer Hospital of Qinghai University, No. 29, Tongren Road, West of the City, Xining, 810000, Qinghai, China; Department of Gynecological Oncology, Affiliated Hospital of Qinghai University, Xining, 810000, Qinghai, China; Department of Gynaecology and Obstetrics, The First People’s Hospital of Xining, Xining, 810000, Qinghai, China; Department of Surgery, Affiliated Hospital of Qinghai University, No. 29, Tongren Road, West of the City, Xining, 810000, Qinghai, China; Department of Gynecologic Oncology, Affiliated Hospital of Qinghai University & Affiliated Cancer Hospital of Qinghai University, No. 29, Tongren Road, West of the City, Xining, 810000, Qinghai, China

**Keywords:** bladder cancer, METTL13, PI3K/ATK

## Abstract

Bladder cancer (BC) is the tenth most common tumor worldwide, characterized by high incidence rates and mortality. This study aimed to explore the role of Methyltransferase like 13 (METTL13) in BC cells. J82 and T24 cells were cultured for *in vitro* experiments. Cell viability, migration, and invasion were assessed using CCK-8 and transwell assays. Senescence-associated beta-galactosidase (SA-β-gal) levels were detected using a β-galactosidase staining kit. METTL13 and cell cycle-related protein levels were quantified using RT-qPCR and Western blotting. The results showed that METTL13 was upregulated in BC cells. Silencing METTL13 decreased cell viability, migration, and invasion in BC cells, whereas METTL13 overexpression increased these parameters. Additionally, METTL13 knockdown inhibited the phosphorylation levels of PI3K, AKT, and mTOR. Inhibition of the PI3K/AKT pathway reversed the effects of METTL13 on cell viability, migration, invasion, and cell cycle-related proteins in BC cells. *In vivo* experiments showed that METTL13 knockdown inhibited tumor growth and development. In conclusion, this study demonstrated that METTL13 promoted the malignant behaviors of BC cells through activation of the PI3K/AKT signaling pathway. METTL13 may be a promising therapeutic target for BC in the future.

## Graphical abstract

**Figure:**
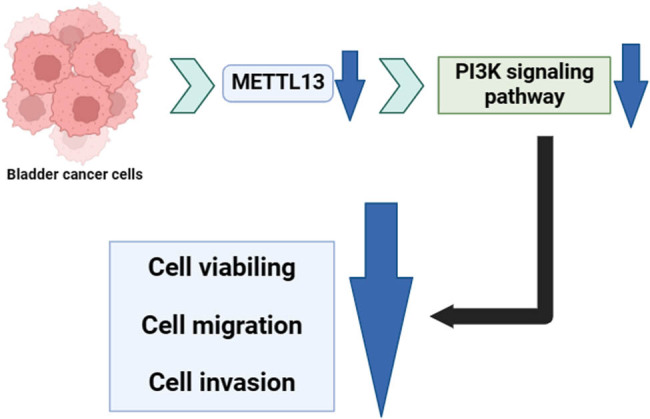

## 1 Introduction

Bladder cancer (BC) ranks as the tenth most common malignancy worldwide, characterized by high incidence rates and mortality [1]. Approximately 90% of the BC cases are urothelial carcinomas, with roughly 75% being non-muscle-invasive, and 25% presenting as muscle-invasive or metastatic BC [2,3]. The postoperative recurrence rate for non-muscle-invasive BC can reach up to 70%, potentially progressing to muscle-invasive disease. BC carries a poor prognosis, with a 5-year overall survival rate of less than 50% [4]. Given the high recurrence rate following surgical resection, there is an urgent need to develop new therapies based on the unique molecular background of BC.

METTL13, located at 1q24.3, was initially identified from rat brain as a suppressor of nuclear apoptosis [5]. Subsequent research has demonstrated that immunogenic METTL13 plays a role in the occurrence of multiple diseases, including various cancers [5,6]. In liver [7], pancreatic [8], gastric [9], and breast cancer [10], METTL13 is highly expressed and is associated with poor patient survival. In recent years, due to two distinct seven-β-strand methyltransferase (MTase) structural domains (MT13-C and MT13-N), METTL13 methylates the N-terminus and Lys55 of eEF1A, respectively, facilitating the synthesis of specific amino acids with high specificity, which contributes to a pro-cancer effect and is ubiquitous in mammalian cells and tissues [6,11,12]. METTL13 can directly or indirectly activate multiple molecular signaling pathways, contributing to the development of various diseases, including those involving KRAS [6]. Although METTL13 has been demonstrated to be an important oncogene in various cancers, one study also confirmed its tumor-suppressive function in renal cell carcinoma [13]. These studies indicate that METTL13 exhibits specificity across different types of tumors. As a potential target for triggering various cancer progressions, it is necessary to fully explore the potential mechanisms of METTL13 in tumor development. However, to date, the specific mechanisms of METTL13 in BC have not yet been elucidated.

The phosphatidylinositol 3-kinase (PI3K)/AKT pathway plays a crucial role in cell growth, proliferation, and survival. The PI3K/AKT signaling pathway is upregulated in many types of cancer [14], including BC [15]. Based on promising preclinical findings, inhibitors targeting the PI3K/AKT signaling pathway are currently considered as therapeutic strategies for BC, either as monotherapies or in combination with cytotoxic drugs [16,17].

Current therapeutic options for BC include surgery, chemotherapy, immunotherapy, and bacillus Calmette-Guérin therapy [18]. While these treatments have improved outcomes, they often come with significant side effects and do not prevent recurrence in many cases. For example, the use of checkpoint inhibitors in advanced BC has shown benefits, but response rates are limited, and resistance can develop over time [19]. While surgical resection remains the gold standard for early-stage disease, the high recurrence rate and the development of resistance to chemotherapy and immunotherapy highlight the necessity for novel therapeutic targets. Targeting METTL13 in BC cells, specifically through its interaction with the PI3K/AKT signaling pathway, represents a promising approach to address these challenges. By understanding how METTL13 influences malignancy, we aim to develop more effective and personalized therapies that can overcome the limitations of current treatment modalities.

Through bioinformatics analysis, we found that METTL13 was closely related to the activation of the PI3K/AKT signaling pathway. Therefore, this study aimed to explore the role of METTL13 in BC progression both *in vitro* and *in vivo*. We hypothesized that METTL13 might promote BC development through the activation of the PI3K/AKT signaling pathway.

## 2 Materials and methods

### 2.1 Bioinformatics analysis

Genes positively correlated with METTL13 in BC were analyzed using the UALCAN online database (https://ualcan.path.uab.edu/index.html) and the top 25 genes were listed. Then these METTL13-related genes were used for kyoto encyclopedia of genes and genomes (KEGG) pathway enrichment analysis (https://david.ncifcrf.gov/). The result was represented as a bubble chart.

### 2.2 Cell culture and treatment

Human ureteral epithelial cells (SV-HUC-1) and BC cell lines, including J82 and T24, were provided by Procell Life Science Co., Ltd. (Wuhan, China). SV-HUC-1 cells were cultured in Ham’s F-12K medium (MacGene, China) supplemented with 10% fetal bovine serum (FBS) (Gibco, MA, USA). J82 and T24 cells were cultured in RPMI-1640 medium (Gibco, MA, USA) supplemented with 10% FBS. All cells were maintained in a humidified atmosphere containing 5% CO_2_ at 37°C. To inhibit the PI3K/AKT signaling pathway, the cells were treated with 20 μM LY294002 for 3 days [20].

The short hairpin RNA targeting METTL13 (sh-METTL13), METTL13 overexpressing plasmids (METTL13), and their respective negative controls (sh-NC and vector) were synthesized by GenePharma (Shanghai, China). Transfection was performed when the cells reached approximately 90% confluence using Lipofectamine 3000 (Thermo Fisher Scientific, MA, USA) according to the manufacturer’s instructions. The transfection efficiency was assessed by performing real-time quantitative PCR (RT-qPCR) 24–48 h post-transfection.

### 2.3 RT-qPCR

Total RNA was extracted from BC cells using TRIzol reagent (Thermo Fisher Scientific, USA). RNA was reverse-transcribed using PrimeScript™ RT Master Mix (Takara, Japan) and analyzed via RT-qPCR using SYBR Premix Ex Taq™ (Takara, Japan). Relative mRNA expression level was calculated by the 2^−ΔΔCt^ method using GAPDH as the internal control. The primers used in this study are exhibited in Table 1.

**Table 1:** Primer sequences

Gene	Primer	Sequence (5′–3′)	Product size (bp)	Annealing temp (°C)	GenBank
METTL13	Forward	CAGGAGGTTGATTACAGTGGC	91	60.5	NM_014955
	Reverse	CTCCATGACTCTAGCCGACA			
GAPDH	Forward	GGAGCGAGATCCCTCCAAAAT	197	61.6	NM_001256799
	Reverse	GGCTGTTGTCATACTTCTCATGG			

### 2.4 Assessment of cell viability

Cell viability was evaluated by CCK-8 assay. J82 and T24 cells were seeded into 96-well plates (5,000 cells/well). After treatment, 10 μL of CCK-8 reagent (Beyotime, Shanghai, China) was added to each well and incubated with the cells for 1–2 h. Absorbance was measured at 450 nm using a spectrophotometer (Shimadzu, Japan).

### 2.5 Transwell assay

Transwell chambers (8 μm pore size; Corning, Corning, NY, USA) with or without Matrigel were used to assess cell invasion and migration. Cell suspension in serum-free dulbecco's modified eagle medium (DMEM) was added in the top chambers, and serum-containing DMEM was added in the bottom chambers. The cells were cultured at 37°C with 5% CO_2_ for 24 h. After culturing, the invaded and migrated cells were fixed with 4% paraformaldehyde and stained with crystal violet for 20 min. The stained cells were viewed under a light microscope.

### 2.6 Western blot

Cells were lysed using radio immunoprecipitation assay lysis buffer (Beyotime, Shanghai, China) on ice to obtain protein extracts. Protein samples were subjected to SDS-PAGE for separation. The separated proteins were then transferred onto polyvinylidene fluoride membranes (Millipore, USA). The membranes were blocked with 5% non-fat dry milk for 2 h. Next, the membranes were incubated with primary antibodies against cyclin A (1:1,500), cyclin B1 (1:1,500), CDK2 (1:1,500), and GAPDH (1:3,000) overnight at 4°C. Subsequently, the membranes were incubated with the appropriate secondary antibody (1:5,000) for 2 h at room temperature. All antibodies were purchased from Abcam (USA). Protein expression was detected using an enhanced chemiluminescence kit (Beyotime, Shanghai, China). The levels of target proteins were normalized using GAPDH as an internal reference.

### 2.7 Determination of SA-β-Gal activity

The cells were inoculated into 6-well plates with 1 × 10^5^ cells/well and incubated for 48 h. Staining was performed using a β-galactosidase staining kit. First, the cells were washed with PBS, followed by fixation with 1 mL of staining fixative solution and incubation in a 37°C and 5% CO_2_ incubator for 15 min. After fixation, the cells were washed three times with PBS. Subsequently, a staining solution consisting of Solution A (10 μL/well), Solution B (10 μL/well), Solution C (930 μL/well), and X-Gal solution (50 μL/well) was added. Then the cells were incubated in a CO_2_-free incubator overnight. Finally, the cells were photographed with an inverted fluorescence microscope to assess intracellular SA-β-Gal activity.

### 2.8 *In vivo* experiments

BALB/c nude mice (female, 5 weeks old) were purchased from Charles River (Beijing, China) and randomly divided into two groups: sh-NC and sh-METTL13, with six mice in each group. T24 cells transfected with sh-NC or sh-METTL13 (5 × 10^6^ cells/100 μL) were subcutaneously injected into the flanks of the BALB/c nude mice. All tumor size was measured every week, and the volume of tumors was calculated as the formula: *V* = 0.52 × *a* ^2^ × *b* (*V* stands for the volume of the tumor, *a* is the smallest diameter, and *b* is the largest diameter). After 4 weeks, all mice were euthanized by intraperitoneal injection of 160 mg/kg pentobarbital sodium. The tumor samples were photographed, weighed, and collected for histological examination (H&E staining).


**Ethical approval:** The research related to animal use has been complied with all the relevant national regulations and institutional policies for the care and use of animals, and has been approved by the Ethics Committee of Affiliated Hospital of Qinghai University (approval no. P-SL-2023-463).

### 2.9 Statistical analysis

All experiments were conducted independently three times, and the results were presented as mean ± SD using GraphPad Prism 7.04 software (GraphPad Software, Inc.). Data analysis was performed using SPSS 19.0 software (SPSS, Inc., Chicago, IL, USA) with *t*-tests or one-way ANOVA to compare differences between two or multiple groups. *P* < 0.05 was considered statistically significant.

## 3 Results

### 3.1 METTL13 promoted the cell viability, migration, and invasion of J82 and T24 cells

**Figure 1:**
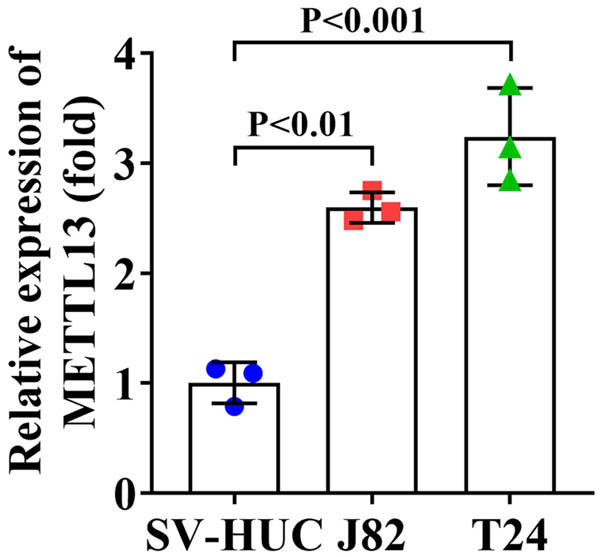
METTL13 was upregulated in BC cells. The METTL13 levels in SV-HUC, J82, and T24 cells were detected by RT-qPCR assay.

**Figure 2:**
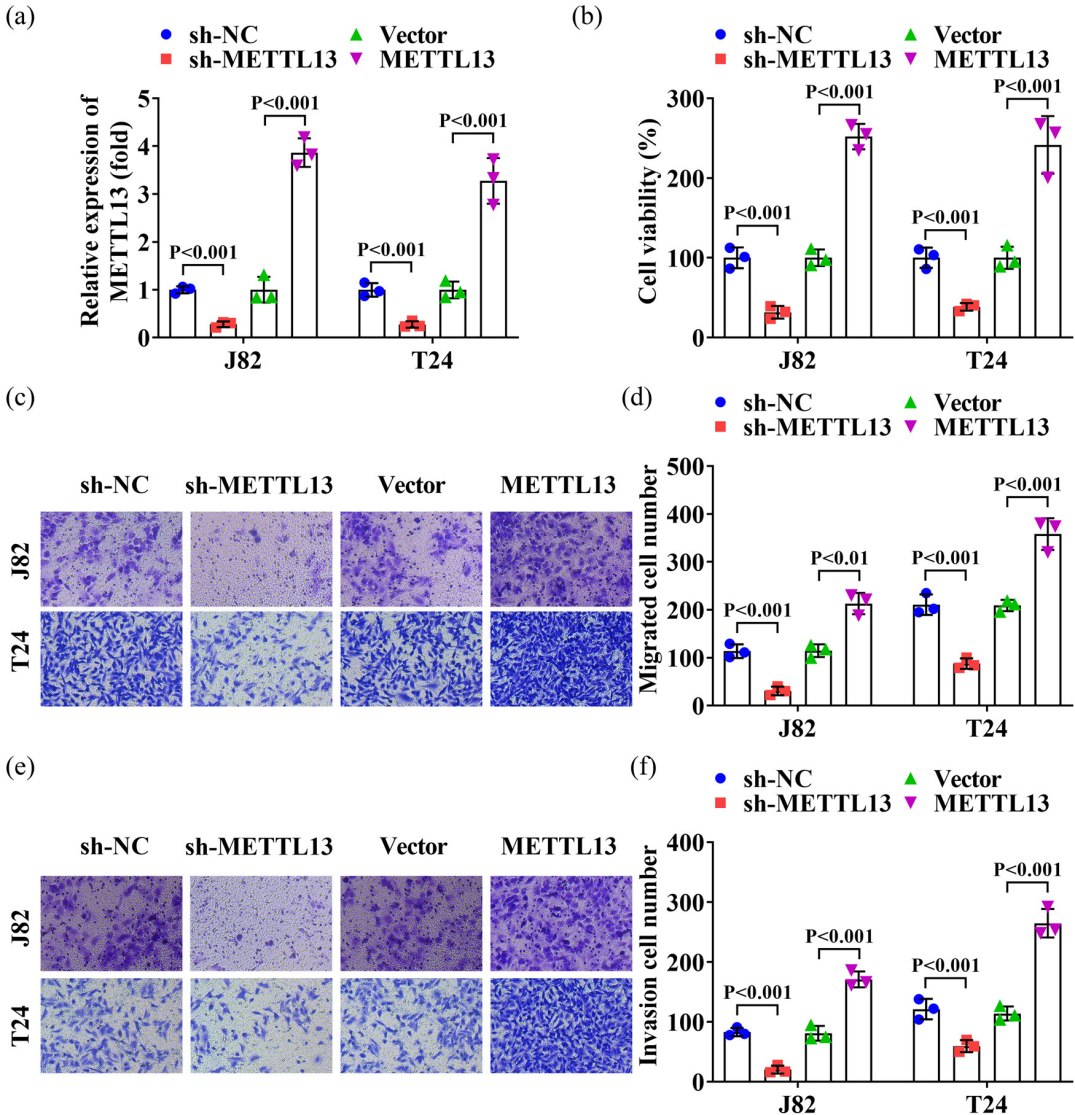
METTL13 promoted the cell viability, migration, and invasion of J82 and T24 cells. (a) Knockout and overexpression efficiency of sh-METTL13 and METTL13 overexpressed plasmids were tested by RT-qPCR. After METTL13 knockdown and overexpression, the cell viability (b), migration (c) and (d) and invasion (e) and (f) abilities of J82 and T24 cells were detected by CCK-8 and Transwell assay. Sh-NC, shRNA negative control. Sh-METTL13, shRNA METTL13. METTL13, METTL13 overexpressed plasmids. Vector, empty overexpressed plasmids.

First, we detected the METTL13 levels in normal bladder epithelial cells and BC cells. The METTL13 levels were significantly higher in J82 and T24 cells compared to normal bladder epithelial cells (Figure 1). Subsequently, J82 and T24 cells were transfected with sh-METTL13 and a METTL13 overexpression vector. RT-qPCR results confirmed that sh-METTL13 transfection decreased the METTL13 levels, while METTL13 overexpression vector transfection increased the METTL13 levels in J82 and T24 cells (Figure 2a). Next, we found that METTL13 knockdown decreased the cell viability (Figure 2b), migration (Figure 2c and d), and invasion (Figure 2e and f) of J82 and T24 cells. Conversely, METTL13 overexpression increased the cell viability (Figure 2b), migration (Figure 2c and d), and invasion (Figure 2e and f) of J82 and T24 cells. These results indicated that METTL13 expression levels were positively associated with the malignant behavior of BC cells.

### 3.2 METTL13 knockdown inhibited the BC development *in vivo*

**Figure 3:**
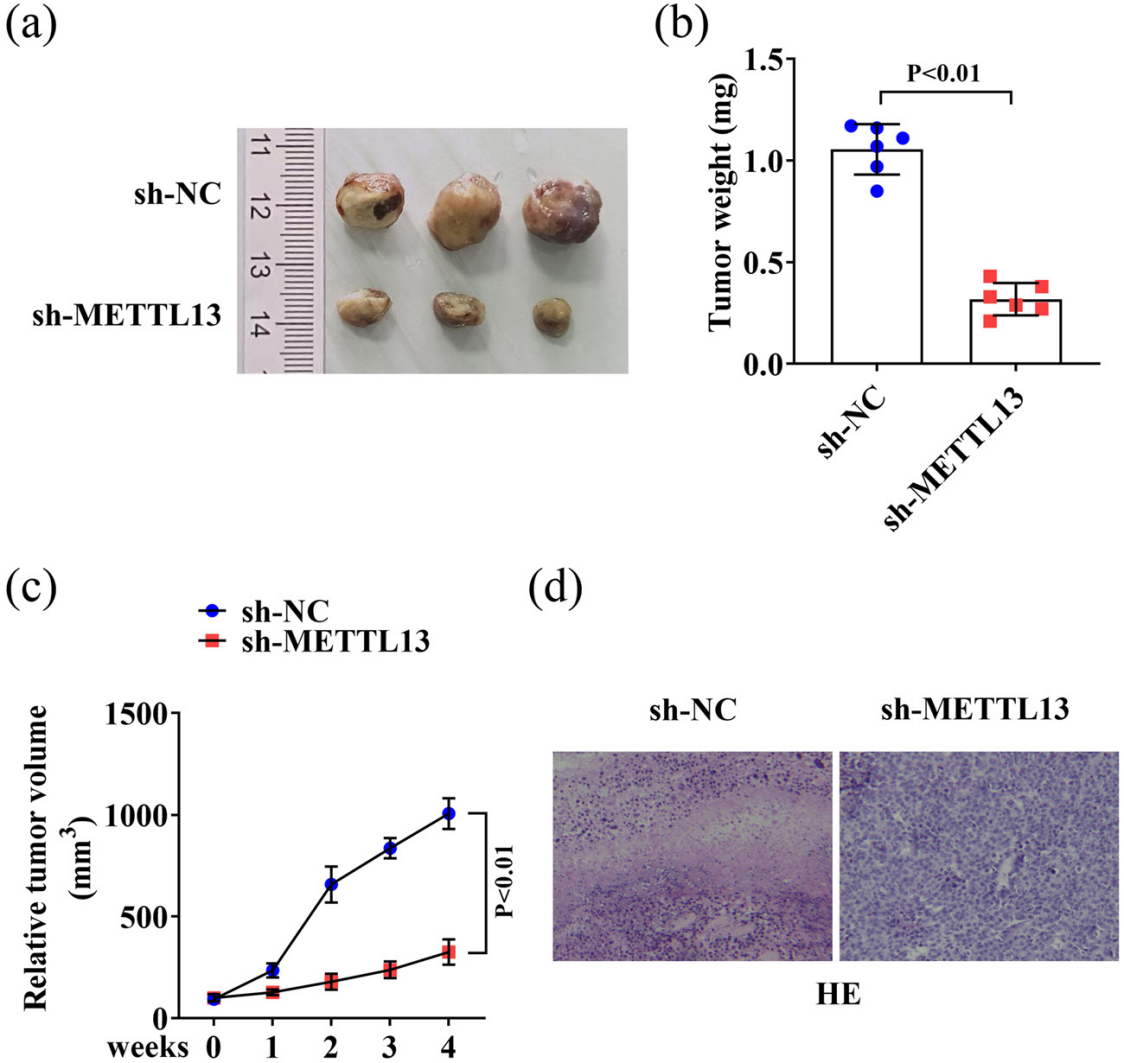
METTL13 knockdown inhibited the BC development *in vivo*. (a) The images of the tumor samples in the sh-NC and sh-METTL13 mice. (b) The weight (b) and volume (c) of the tumor samples in the sh-NC and sh-METTL13 mice. (d) HE staining of the tumor samples in the sh-NC and sh-METTL13 mice. Sh-NC, shRNA negative control. Sh-METTL13, shRNA METTL13. HE, hematoxylin-eosin.

To explore the role of METTL13 downregulation in tumor growth *in vivo*, stable sh-METTL13-transfected T24 cells and corresponding control cells were subcutaneously injected into the flanks of nude mice. The results showed that knockdown of METTL13 in T24 cells significantly decreased tumor growth, as evidenced by reduced tumor weight (Figure 3a and b) and volume (Figure 3c) *in vivo* compared to the control cells. Histological examination using H&E staining revealed that the tumor tissue in the sh-NC group displayed more mitotic figures with no obvious signs of necrosis. In contrast, the sh-METTL13 group showed pyknotic or fragmented nuclei with fewer mitotic figures, and focal tumor cell necrosis was observed (Figure 3d). These results suggested that METTL13 may play a critical role in BC carcinogenesis.

### 3.3 METTL13 was closely related to PI3K/AKT signaling pathway

**Figure 4:**
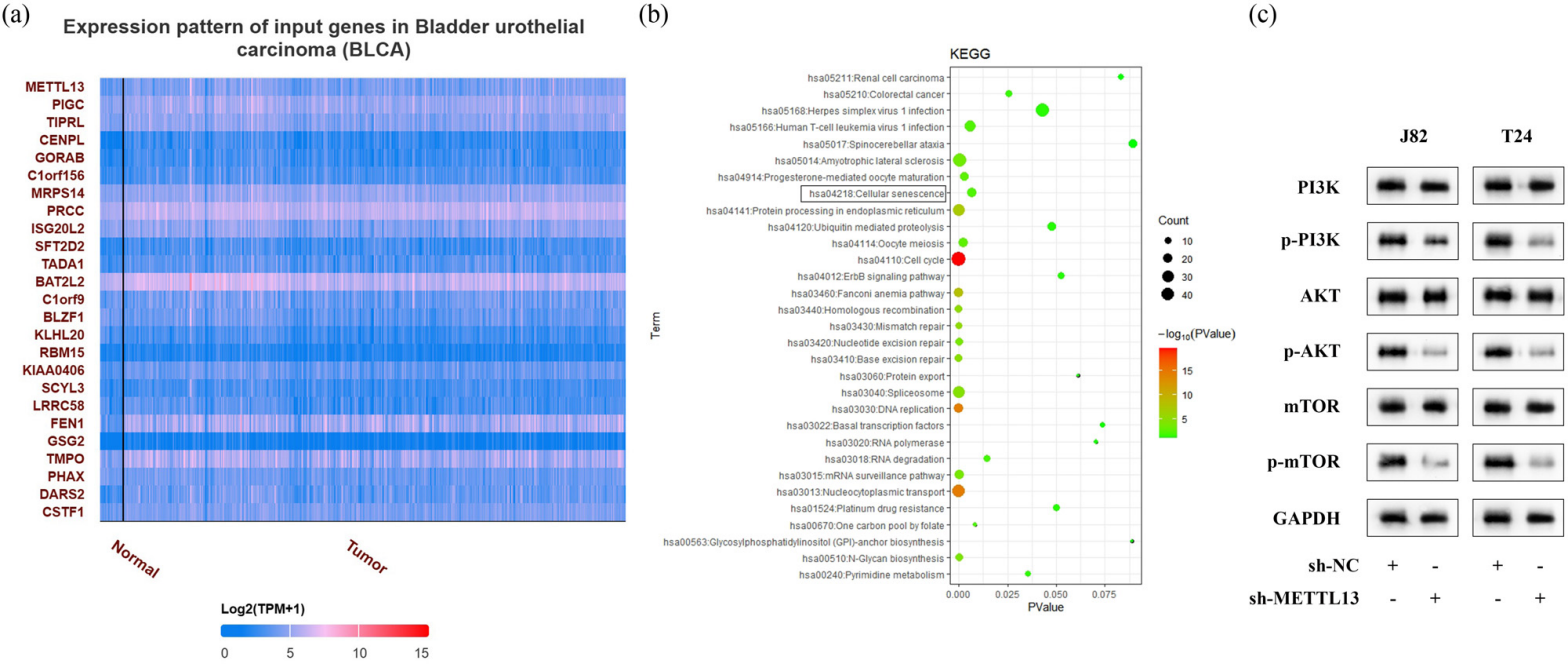
METTL13 was closey related to PI3K/AKT signaling pathway. (a) Genes positively correlated with METTL13 in BC were analyzed using the UALCAN online database and the top 25 genes were listed. (b) KEGG analysis of Genes positively correlated with METTL13 in BC. (c) Protein levels of PI3K, AKT, and mTOR in the METTL13 knockdown J82 and T24 cells were detected by Western blot analysis. Sh-NC, shRNA negative control. Sh-METTL13, shRNA METTL13.

Next, through the UALCAN online database, we obtained genes positively correlated with METTL13 in BC and listed the top 25 genes (Figure 4a). Through KEGG pathway analysis, these METTL13-related genes were found to be enriched in 31 key signaling pathways, including the cellular senescence pathway (Figure 4b). In the process of cellular senescence, the PI3K/AKT signaling pathway played a key role [21]. We then found that METTL13 knockdown significantly decreased the protein levels of p-PI3K, p-AKT, and p-mTOR in J82 and T24 cells (Figure 4c), indicating that METTL13 knockdown inhibited the activation of the PI3K/AKT signaling pathway.

### 3.4 LY294002 treatment reversed the role of METTL13 overexpression in the J82 and T24 cells

**Figure 5:**
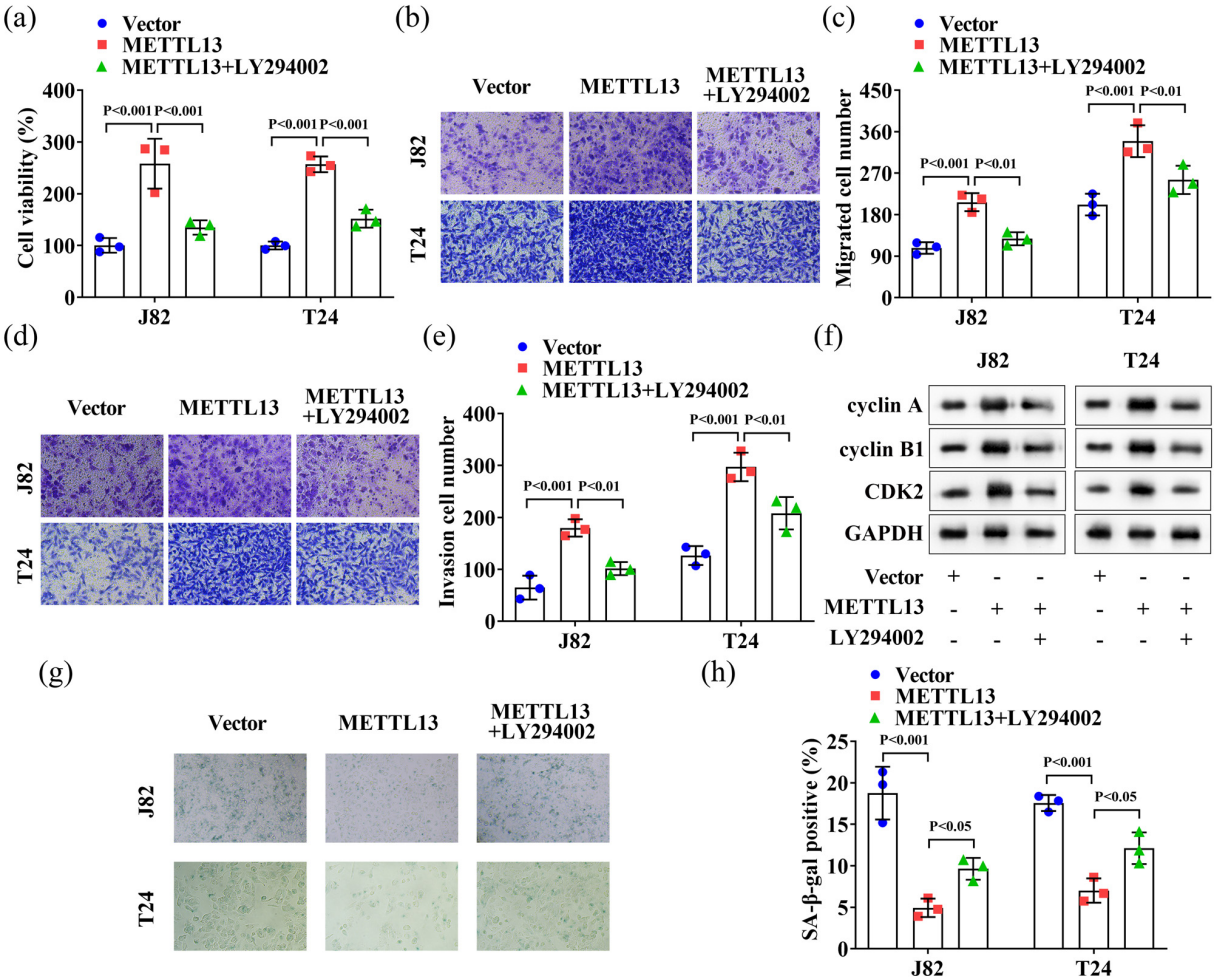
LY294002 treatment reversed the role of METTL13 overexpression in the J82 and T24 cells. (a) The J82 and T24 cells were treated with METTL13 overexpressed plasmids and PI3K/AKT pathway inhibitor (LY294002). Then the cell viability (a), migration (b) and (c) and invasion (d) and (e) abilities of J82 and T24 cells were detected by CCK-8 and Transwell assays. (f) The protein levels of cyclin A, cyclin B1, and CDK2 of J82 and T24 cells were detected by Western blot analysis. (g) and (h) The SA-β-gal levels of J82 and T24 cells were detected by β-galactosidase staining kit. METTL13, METTL13 overexpressed plasmids. Vector, empty overexpressed plasmids. LY294002, PI3K/AKT pathway inhibitor. SA-β-gal, senescence-associated β-galactosidase.

LY294002 is a known inhibitor of the PI3K/AKT pathway [22]. Here, we used LY294002 to treat METTL13-overexpressing J82 and T24 cells to explore whether METTL13 participates in regulating cell viability, migration, and invasion by modulating the PI3K/AKT signaling pathway. After METTL13 overexpression, the cell viability (Figure 5a), migration (Figure 5b and c), and invasion (Figure 5d and e) of the J82 and T24 cells were significantly increased. Treatment with LY294002 significantly decreased the cell viability (Figure 5a), migration (Figure 5b and c), and invasion (Figure 5d and e) abilities. Furthermore, Western blot analysis indicated that METTL13 overexpression significantly increased the protein levels of cyclin A, cyclin B1, and CDK2 in J82 and T24 cells, and LY294002 treatment significantly decreased these levels (Figure 5f). Additionally, METTL13 overexpression significantly decreased the SA-β-gal levels in J82 and T24 cells, whereas LY294002 treatment significantly increased these levels (Figure 5g and h). These results indicated that METTL13 promoted the malignant behaviors of BC cells through activation of the PI3K/AKT signaling pathway.

## 4 Discussion

In the present study, we found that METTL13 was upregulated in BC cells. Mechanistically, we demonstrated that METTL13 knockdown inhibited the malignant behaviors of BC cells, while METTL13 overexpression promoted these behaviors. Importantly, inhibiting the PI3K/AKT signaling pathway reversed the role of METTL13 in BC cells.

METTL13, a member of the N-terminal protein MTase family, has been shown by numerous studies to promote the development of various cancers [23]. METTL13 is involved in cell apoptosis, stem cell biology, and various cellular signaling and metabolic pathways [11]. In head and neck squamous cell carcinoma, METTL3 was significantly upregulated and negatively associated with clinical prognosis. METTL13 overexpression markedly promoted epithelial–mesenchymal transition and induced the malignant behaviors of cancer cells [24]. In gastric cancer, Wu et al. [9] confirmed that high expression of METTL13 was closely associated with tumor size and T classification. METTL13 knockdown inhibited gastric cancer cell proliferation and metastasis both *in vivo* and *in vitro*. However, conversely, Liu et al. [13] found that METTL13 was downregulated in clear cell renal cell carcinoma tissues. Overexpression of METTL13 decreased the proliferation, migration, and invasion of clear cell renal cell carcinoma cells and inhibited the development of epithelial–mesenchymal transition. We speculated that the contradictory roles of METTL13 in cancers may lie in its different modification mechanisms across specific organs. Here, we also found that METTL13 was highly expressed in BC cells. METTL13 overexpression promoted the cell viability, migration, and invasion of BC cells. In addition, the results of our *in vivo* experiments showed that METTL13 knockdown inhibited the growth of BC tumors. Our findings further confirmed the oncogenic role of METTL13 in BC.

Subsequently, through the UALCAN online database, we identified that genes positively correlated with METTL13 in BC. The KEGG analysis results showed that these genes were enriched in 31 key signaling pathways. Among these pathways, the cellular senescence pathway has a particularly close relationship with BC development. On the other hand, during the cellular senescence process, the PI3K/AKT signaling pathway plays an important role [25]. Numerous studies have demonstrated that activation of the PI3K/AKT signaling pathway accelerated the cell cycle, promotes cell growth, and inhibits cellular senescence [26,27]. In the context of BC development, many studies have shown that inhibiting the PI3K/AKT signaling pathway prevents the malignant behaviors of cancer cells and reduces the growth rate of tumor tissues. For example, Chi et al. [15] found that TEAD4, a member of the TEA domain (TEAD) transcription factors family, promoted the metastasis of cancer cells and the development of epithelial–mesenchymal transition via the PI3K/AKT pathway. Zhu et al. [28] confirmed that the METTL3/YTHDF1 axis, mediating m6A methylation, regulated the proliferation and cisplatin sensitivity of BC cells through targeting the PI3K/AKT pathway. Here, we found that METTL13 knockdown inhibited the PI3K/AKT signaling pathway. Interestingly, treatment with LY294002, a PI3K/AKT pathway inhibitor, reversed the effects of METTL13 overexpression on the cell viability, migration, and invasion of BC cells. In addition, considering the relationship between METTL13 and the cellular senescence pathway, we further analyzed the effect of METTL13 on the expression levels of cell cycle-related proteins and SA-β-gal levels. We found that METTL13 overexpression significantly increased the protein levels of cyclin A, cyclin B1, and CDK2 and decreased SA-β-gal levels in BC cells, while LY294002 treatment significantly reversed these changes. These results suggested that METTL13 may induce malignant cell behavior and ultimately exacerbate tumor progression by activating the PI3K/AKT signaling pathway.

However, there are some limitations to this study. Due to constraints in hospital resources, we were unable to obtain sufficient clinical samples to conduct comprehensive clinical studies. Furthermore, additional research is needed to elucidate the specific mechanism by which METTL13 regulates the PI3K/AKT signaling pathway. Although our study did not involve a clinical trial, the potential for future clinical applications is an important consideration. Given the involvement of METTL13 in the PI3K/AKT signaling pathway, drugs that target this pathway, such as PI3K inhibitors (e.g., alpelisib) [29] and AKT inhibitors (e.g., ipatasertib) [30], could be evaluated for their potential to indirectly affect METTL13 activity. Additionally, efforts to identify direct inhibitors of METTL13 could be pursued in preclinical models, paving the way for clinical trials to assess their safety and efficacy in BC patients. Although specific therapies targeting METTL13 have not yet been developed, our findings may pave the way for future research into such targeted therapies. We plan to conduct further investigations into the sensitivity of METTL13 to existing medications and explore potential synergies with other therapeutic strategies.

In conclusion, we have identified a novel METTL13/PI3K/AKT signaling pathway that contributes to BC cell growth, migration, and invasion. METTL13 may emerge as a promising therapeutic biomarker for BC in the future.
